# Possibilities of Using Specific Jominy Distance in ANN Models for Predicting Low-Alloy Steels’ Microstructure

**DOI:** 10.3390/ma18030564

**Published:** 2025-01-26

**Authors:** Tea Marohnić, Sunčana Smokvina Hanza, Dario Iljkić, Robert Basan

**Affiliations:** University of Rijeka, Faculty of Engineering, Vukovarska 58, 51000 Rijeka, Croatia; tmarohnic@riteh.uniri.hr (T.M.); suncana.smokvinahanza@riteh.uniri.hr (S.S.H.); dario.iljkic@riteh.uniri.hr (D.I.)

**Keywords:** steels for quenching and tempering, case hardening steels, quenching, microstructure, ferrite, pearlite, bainite, martensite, artificial neural networks, Jominy test

## Abstract

Understanding the volume fractions of microstructure constituents such as ferrite, pearlite, bainite, and martensite in low-alloy steels is critical for tailoring mechanical properties to specific engineering applications. To address the complexity of these relationships, this study explores the use of artificial neural networks (ANNs) as a robust tool for predicting these microstructure constituents based on alloy composition, specific Jominy distance, and heat treatment parameters. Unlike previous ANN-based predictions that rely on the hardness after quenching as an input parameter, this study excludes hardness. The developed model relies on readily available input parameters, enabling accurate estimation of microstructure composition prior to heat treatment, which significantly improves its practicality for process planning, optimization, and reducing trial-and-error on industrial applications. Three different input configurations were tested to evaluate the predictive capabilities of ANNs, with results showing that the use of specific Jominy distance as an input variable enhances model performance. Furthermore, the findings suggest that specific Jominy distance could serve as a practical alternative to detailed chemical composition data in industrial applications. The predictions for ferrite, pearlite, and martensite were more accurate than those for bainite, which can be attributed to the complex nature of bainite formation.

## 1. Introduction

Quenching is a frequently used heat treatment process, usually involving the rapid cooling of steel from its austenitizing temperature. The primary goal of quenching is to achieve the desired mechanical properties by forming martensite, or by stabilizing high-temperature phases in alloys. However, the achieving of these goals must be balanced with minimizing deformation and residual stresses, which remain significant challenges due to the complex nature of quenching. For example, in [1], strain and stress evolution during the transformation of coupled bainite and martensite is modeled, and mechanism-based cooling strategies to design tailored residual stress configurations in multiphase steel tubes are proposed. In [2], authors studied the quenching process of AISI 4340 steel samples using a FEM model to predict dimensional behavior, internal stress evolution, and mechanical properties. In [3], the impact of titanium microalloying on the quenching residual stress of H13 steel is investigated to analyze the temperature and stress evolution of Ti microalloying H13 steel.

The prediction of microstructure transformations is essential for modeling and optimizing the quenching process [4,5]. Such predictions are not only important for estimating mechanical properties but also for anticipating stresses and strains generated during quenching [6]. The traditional approaches to modeling these transformations rely on mathematical models; however, these models often involve simplifying assumptions that limit their accuracy [7,8,9]. Furthermore, the theory of microstructural transformations in steels is still a subject of controversy [10]. As a result, numerical simulations based on such models may deviate from experimental observations and could be improved.

Advanced computational tools for prediction, such as artificial neural networks (ANNs), have shown immense promise in addressing these challenges. ANNs, a subset of machine learning, are designed to model nonlinear and complex relationships by learning directly from data. This capability makes them particularly well suited not only for predicting microstructure transformations in steels, but also for forecasting their mechanical properties, such as hardness, tensile strength, and yield strength.

For example, Sitek and Trzaska (2021) reviewed the practical aspects of designing and using artificial neural networks in materials engineering, emphasizing the importance of dataset quality and ANN architecture in accurately predicting steel properties under varying processing conditions [11]. Patel et al. (2024) developed an integrated model combining artificial neural networks and genetic algorithms to predict the relationships between chemical composition, microstructure, and mechanical properties in additively manufactured steels, enabling more precise tailoring of material characteristics for specific applications [12].

The aim of this research was to explore the application of artificial neural networks in predicting the volume fractions of microstructure constituents—ferrite, pearlite, bainite, and martensite—with a particular focus on steels for quenching and tempering as well as case-hardening steels designed for carburizing. The research builds upon the findings presented in the study focused on developing ANN models to predict the hardness of low-alloy steels when detailed chemical composition is unknown [13].

Steels for quenching and tempering as well as case-hardening steels designed for carburizing are hypoeutectoid low-alloy steels, containing less than ~0.8 wt.% carbon, and alloying elements, including carbon, with a total content of up to ~5.0 wt.%. Steels intended for heat treatment, which enables the achievement of desired mechanical properties, should exhibit high hardenability to ensure attaining high strength and toughness throughout the entire heat-treated component.

The developed ANN-based prediction of steels microstructure is based on readily available input parameters, including detailed chemical composition, the specific Jominy distance, and heat treatment parameters, all of which can be determined before the heat treatment process. Unlike previous ANN-based models that rely on post-quenching hardnesses as an input parameter [14,15], this approach excludes hardness since it is not known before the heat treatment. Moreover, excluding hardness avoids the direct correlation between hardness and microstructure. This methodology enables the development of a more versatile predictive model that can estimate microstructure composition prior to performing the heat treatment, enhancing its practical utility in process planning and optimization.

## 2. Materials and Data

### 2.1. Materials

Table 1 presents the chemical composition of studied steels for quenching and tempering as well as case-hardening steels designed for carburizing. Quenching, tempering, and case-hardening are essential heat treatment processes for steels, enhancing their mechanical properties for various industrial applications. Quenching increases hardness and wear resistance, tempering reduces brittleness and improves toughness, and case-hardening creates a hard, wear-resistant surface with a tough core. Together, these processes achieve an optimal balance for durability and performance, making steel suitable for high-stress and wear-intensive conditions. In Table 1, rows No. 1–8 correspond to case-hardening steels, while rows No. 9–24 refer to steels intended for quenching and tempering. Steels marked with superscript “1” have a higher carbon content compared to the standard carbon content in steels.

The primary alloying elements in these steels are carbon, silicon, manganese, chromium, molybdenum, and nickel. Manganese, chromium, molybdenum, and nickel improve the hardenability of steels, while silicon increases yield strength and further enhances hardenability when combined with manganese or molybdenum [16].

**Table 1:** The chemical composition of the studied steels (balance Fe) [13,17].

Data No.	Designation (DIN)	Chemical Composition, wt.%										
		C	Si	Mn	P	S	Al	Cr	Cu	Mo	Ni	V
1.	Ck15	0.15	0.22	0.41	0.021	0.024	<0.005	0.06	0.15	-	0.06	-
2.	Ck15 ^1^	0.30	0.29	0.39	0.012	0.026	0.003	0.12	0.215	-	-	-
3.	16MnCr5	0.16	0.22	1.12	0.030	0.008	0.015	0.99	-	0.02	0.12	0.01
4.	15CrNi6	0.13	0.31	0.51	0.023	0.009	0.010	1.50	-	0.06	1.55	<0.01
5.	20MoCr4 ^1^	0.28	0.30	0.66	0.018	0.011	0.049	0.56	0.18	0.44	0.15	-
6.	20MoCr4 ^1^	0.57	0.30	0.66	0.018	0.011	0.049	0.56	0.18	0.44	0.15	-
7.	25MoCr4 ^1^	0.31	0.20	0.67	0.017	0.022	0.034	0.50	-	0.45	0.11	-
8.	20NiMoCr6 ^1^	0.28	0.15	0.62	0.015	0.020	0.015	0.47	-	0.48	1.58	-
9.	Ck45	0.44	0.22	0.66	0.022	0.029	-	0.15	-	-	-	0.02
10.	37MnSi5	0.38	1.05	1.14	0.035	0.019	-	0.23	-	-	-	0.02
11.	42MnV7	0.43	0.28	1.67	0.021	0.008	-	0.32	0.06	0.03	0.11	0.10
12.	34Cr4	0.35	0.23	0.65	0.026	0.013	-	1.11	0.18	0.05	0.23	<0.01
13.	34Cr4	0.36	0.29	0.69	0.011	0.014	-	1.09	0.12	0.07	0.08	0.01
14.	41Cr4	0.44	0.22	0.80	0.030	0.023	-	1.04	0.17	0.04	0.26	<0.01
15.	41Cr4	0.41	0.25	0.71	0.031	0.024	-	1.06	0.17	0.02	0.22	<0.01
16.	36Cr6	0.36	0.25	0.49	0.021	0.020	-	1.54	0.16	0.03	0.21	<0.01
17.	25CrMo4	0.22	0.25	0.64	0.010	0.011	-	0.97	0.16	0.23	0.33	<0.01
18.	34CrMo4	0.30	0.22	0.64	0.011	0.012	-	1.01	0.19	0.24	0.11	<0.01
19.	42CrMo4	0.38	0.23	0.64	0.019	0.013	-	0.99	0.17	0.16	0.08	<0.01
20.	50CrMo4	0.50	0.32	0.80	0.017	0.022	-	1.04	0.17	0.24	0.11	<0.01
21.	50CrMo4	0.46	0.22	0.50	0.015	0.014	-	1.00	0.26	0.21	0.22	<0.01
22.	27MnCrV4	0.24	0.21	1.06	0.014	0.020	-	0.79	0.17	0.02	0.18	<0.01
23.	50CrV4	0.55	0.22	0.98	0.017	0.013	-	1.02	0.07	-	0.01	0.11
24.	50CrV4	0.47	0.35	0.82	0.035	0.015	-	1.20	0.14	-	0.04	0.11

^1^ Higher carbon content relative to standard carbon content in steels.

### 2.2. Input Variables and Data

The volume fractions of steel microconstituents, namely martensite, bainite, and ferrite-pearlite mixture, primarily depend on the chemical composition and the temperature evolution during the heat treatment. For this reason, the prediction of the volume fractions of steel microconstituents using ANNs was designed to utilize 10 input variables: the main alloying elements, the heat treatment parameters, and the specific Jominy distance, as detailed in Table 2. In line with this, an original dataset was generated. All input data were derived from experimental results obtained from the literature [17]. Based on these input data, a model for microstructure prediction has been established, which can be used even for steels whose experimental data are not known or available in the literature.

Different alloying elements suppress the diffusional pearlitic and bainitic transformations in steels to varying degrees, significantly influencing the volume fractions of microconstituents.

The temperature variation at any point in the component is the key driving force for phase transformations. Higher cooling rates during the cooling of steel from the austenitizing temperature result in a higher volume fraction of hard martensite, whereas lower cooling rates lead to a higher volume fraction of the softer ferrite-pearlite mixture. From this, it can be concluded that the volume fractions of these microconstituents are primarily determined by the cooling rate of the steel, which is effectively defined by the cooling time from the austenitizing temperature to 500 °C. This is further supported by the established relationship between the as-quenched hardness of steel and the cooling time from 800 °C to 500 °C, as widely recognized in the literature [17] and practice.

Furthermore, heat treatment parameters, such as the austenitizing temperature and the austenitizing time, are also included as input parameters, as higher austenitizing temperatures and longer austenitizing times promote austenite grain growth and enhance the solubility of carbon and other alloying elements in austenite, thereby influencing the kinetic of austenite decomposition.

The microstructure achieved through steel quenching also depends on the steel’s hardenability. Therefore, in addition to the chemical composition, the specific Jominy distance was included as an input variable. This distance is directly related to the hardenability of steel, depending mostly on alloying elements, and corresponds to the Jominy distance at which 50% of the microstructure consists of martensite [13].

## 3. Methods

### Development of Artificial Neural Networks for Prediction of Low-Alloy Steels’ Volume Fractions of Microstructure Constituents

**Figure 1:**
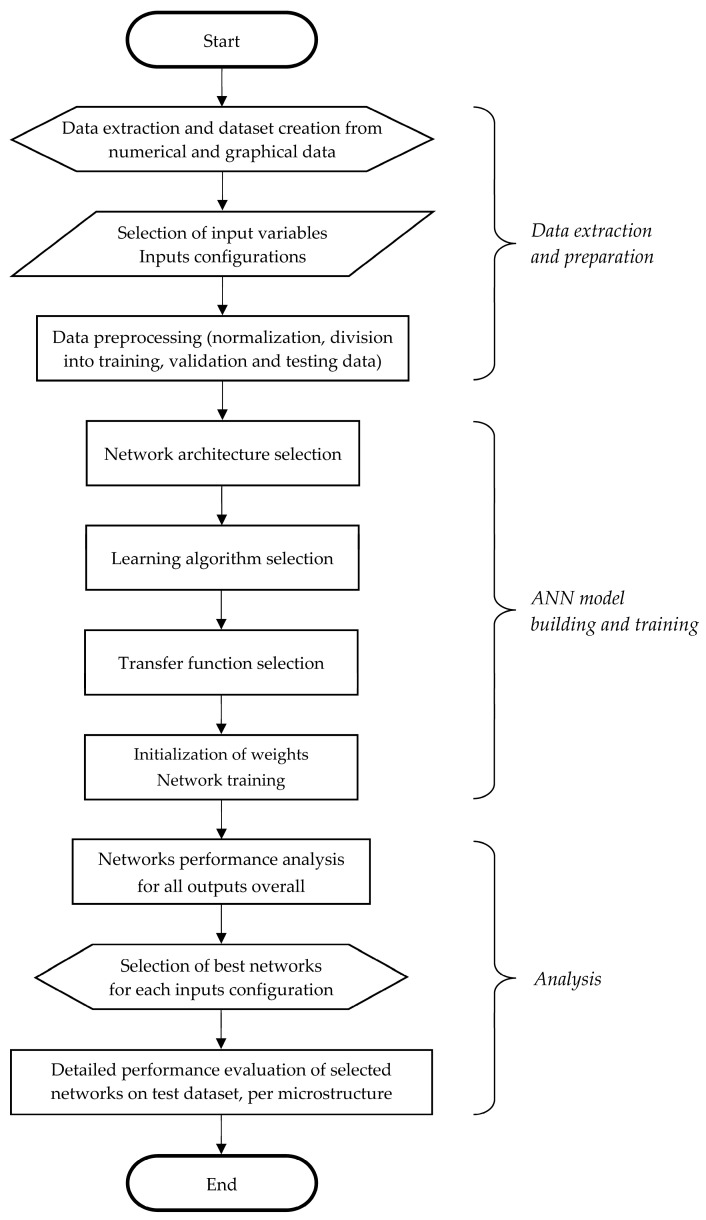
Flow chart of development procedure of ANNs for prediction of low-alloy steels’ volume fraction of microstructure constituents.

In its nature, the prediction of steels volume fractions of microstructure constituents is a regression, i.e., function approximation problem. For such problems, in various research areas including material property prediction, ANNs are being used as versatile, nonlinear computational models inspired by biological neural systems. The proposed procedure of estimating the low-alloy steels volume fractions of microstructure constituents using artificial neural networks, including data extraction and preparation, ANNs model building and training, and analysis of the ANNs performance, is given in the flow chart in Figure 1 and explained in more details in the following paragraphs.

**Figure 2:**
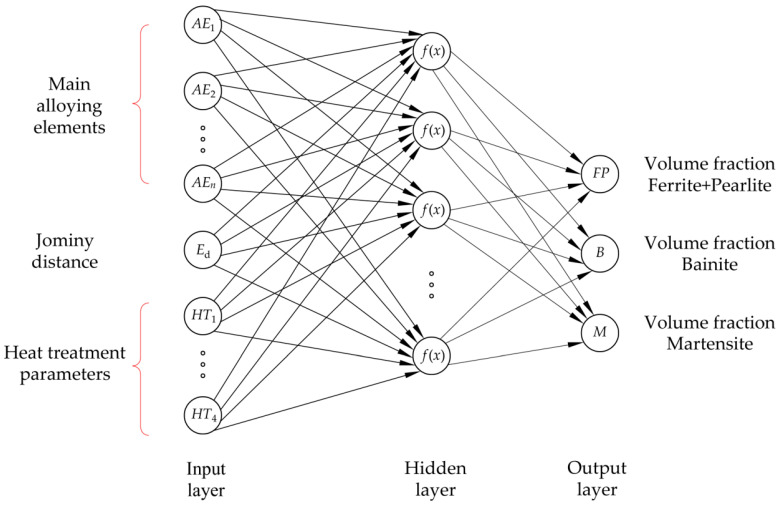
Fully connected multilayer perceptron with one hidden layer.

ANNs consist of an input layer, one or more hidden layers, and an output layer. Input and output layers have one neuron per input and output variable, while the size and the number of hidden layer(s) can vary. In a fully connected ANN, all neurons are interconnected by weights. A fully connected multilayer perceptron with one hidden layer and all input variables relevant for this study (listed in Table 2) is shown in Figure 2.

In this study, i.e., our prediction of steels’ volume fractions of microstructure constituents, several two-layer multilayer perceptrons (MLPs) were developed to estimate the steels microstructure, taking advantage of MLPs’ ability to model complex, nonlinear relationships in the data. According to [18], a two-layer MLP with a *hyperbolic tangent* transfer function in the hidden layer and *linear* transfer function in the output layer is considered a universal approximator and is efficient in solving most regression problems. Due to the dataset being on the smaller side, a single hidden layer, which is appropriate for avoiding overfitting and ensuring generalization with limited data, was chosen. However, a combination of *tansig* and *linear* activation functions did not prove to be adequate for the problem presented in this research. Two conditions should be met when estimating the volume fractions of microstructure constituents. First, estimated values should be non-negative, and second, the sum of all outputs should be equal to 1. In addition to the *linear* transfer function, the *logsig* function was explored for the hidden layer. In the output layer, in addition to the *linear* transfer function, *sigmoid* and *softmax* transfer functions were considered. The final configuration yielded the *logsig* transfer function in the hidden layer and *softmax* in the output layer. The outputs of the *softmax* transfer function can be interpreted as the probabilities associated with each class (or here, the volume fraction of each phase). Each output will fall between 0 and 1, and the sum of the outputs will equal 1 [18]. Although *softmax* is more commonly used in pattern recognition or classification problems, here it aligns with the nature of the research problem.

The *backpropagation* algorithm, which is often used for supervised learning with a multilayer perceptron, is also used in this research. The primary goal of artificial neural network training is to adjust the weights so that the error function is minimized. Here, the *mean square error*, *MSE*, is chosen as the error function. The initialization of weights marks the beginning of the training, followed by the propagation of input signals through the network from the input layer to the output layer (*forward phase*). The forward phase is followed by *backpropagation* (*backward phase*). In backpropagation, error signals which are calculated by comparing predicted (i.e., output) and target values, propagate from the output layer to the input layer. During this phase, the weights are updated iteratively to reduce the error. The process continues until a specified stopping criterion is met.

Different combinations of input variables were considered and used for artificial neural networks development. The first configuration of input variables included the main alloying elements, austenitizing temperature, *T*_a_; austenitizing time, *t*_a_; cooling time to 500 °C, *t*_500_; and specific Jominy distance, *E*_d_. The second configuration omits the specific Jominy distance, while in the third configuration of input variables specific Jominy distance was used as the input variable (instead of the main alloying elements) along with the heat treatment parameters *T*_a_, *t*_a_, and *t*_500_. Input variables for individual configurations are listed in Table 3. Every MLP had three output variables—volume fractions of ferrite-pearlite, bainite, and martensite. This was decided after the initial investigation, where the prediction of each microstructural constituent was performed with a separate ANN. In theory, three ANNs with one output should yield the same result as one ANN with three outputs. However, this did not provide good results in this case, since outputs are strongly interdependent.

For the development and testing of the artificial neural networks with all three configurations of input variables, 423 datasets for 24 steels were used (Supplementary Materials, Table S1). For the purpose of this research, ANNs were developed using the computer software MATLAB R2022b [19].

ANN robustness is ensured by preventing overlearning and overfitting, as well as by evaluating the ANNs’ performances on test data, i.e., those that were not used for ANNs development.

Overlearning was prevented by combining the “growth method” for the determination of the number of neurons in the hidden layer, and early stopping as a principle for improving generalization. Early stopping implies that weights are updated for the training dataset while the error function (mean square error, *MSE*) is calculated for the validation dataset. The training is stopped once the value of *MSE* on the validation dataset reaches a minimum, and then increases for a predefined number of epochs.

The size of the hidden layer, i.e., the maximum number of neurons in the hidden layer, *H*, for which the ANNs with different configurations of input variables were trained, is determined depending on the number of available training equations, *N*_traineq_, the number of input variables, *I*, and the number of output variables, *O*:

(1) $$H\leq \frac{O(N_{traineq}-1)}{I+O+1}.$$

Limiting the maximum number of neurons in the hidden layer, *H,* is important for preventing overfitting. The Levenberg–Marquardt algorithm with early stopping was used for the training of networks with three different combinations of input variables (as listed in Table 3), and hidden layer size from one neuron to *H* neurons (“growth method”). According to [18], this learning algorithm with backpropagation appears to be among the fastest ANN training algorithms for moderate numbers of network parameters. The maximum number of neurons *H* was different for each configuration, in line with Equation (1). Each architecture was trained 10 times with random initial weights and data divisions. In total, 2410 networks were trained—630 for Configuration No. 1, 680 for Configuration No. 2, and 1100 for Configuration No. 3.

Initialization of weights determines the starting point of ANN training and, if this is carried out randomly, for 10 iterations, the odds that said starting point is determined well and that *MSE* will reach the global minimum is increased.

The most important hyperparameters explored for the development of ANNs in this research are given in Table 4. Hyperparameters that were selected for the final ANN configurations are underlined.

The Levenberg–Marquardt algorithm requires data division into training, validation and testing datasets. Using ten trainings per each architecture also ensures that random data division is performed in such a way that these three datasets represent the entire population. Fractions of data assigned to training, validation and testing are commonly set to 0.7/0.15/0.15 for training, validation, and testing datasets, respectively, which is adopted here as well.

If *N* is the total number of datasets used for the development of ANNs, the number of training examples, *N*_train_, is then:

(2) $$N_{train}=0.7N$$

while the number of training equations *N*_traineq_ is:

(3) $$N_{traineq}={O\cdot N}_{train}.$$

The number of training equations *N*_traineq_ was constant for all networks, regardless of the number of input variables and architecture. Output variables were always volume fractions of ferrite and pearlite, bainite, and martensite, which gives the number of outputs, *O* = 3.

The number of unknown weights, *N*_w_, in a fully connected MLP with one hidden layer is:

(4) $$N_{w}=\left(I+1\right)H+(H+1)O.$$

Several limitations should be kept in mind when determining the size of the hidden layer. The number of weights, *N*_w_, should be a lot smaller than the number of training equations, i.e., *N*_w_ << *N*_traineq_, or in extreme cases their difference, the number of degrees of freedom, *N*_dof_, must be greater than zero:

(5) $$N_{dof}=N_{traineq}-N_{w}>0.$$

The number of training equations, *N*_traineq_, should be 4–5 times greater than the number of unknown weights, *N*_w_. For a worst-case scenario with a maximum number of input variables (10, Configuration No. 1), to fulfill these conditions, the maximum feasible number of neurons in hidden layer, *H*, is between 12 and 15.

Since the original data were divided into training, validation, and testing subsets, the regression analysis between target and output values should be performed on each subset individually, as well as on the full dataset. Should the ANN show accurate fitting on the training subset, but poor results on the validation and test subsets, this would indicate overfitting. If training and validation results are good, but the testing results are poor, this could indicate extrapolation [18]. Since ANNs learn by example, they are only reliable if applied to the same data distribution, as was the case with the learning dataset.

The selection of the best performing artificial neural network, for all three configurations and all architectures trained (hidden layer size *H*), was based on the value of coefficients of correlation, *r*, and the value of root mean square error, *RMSE*, for the whole dataset. A useful indicator of model accuracy is root mean square error, *RMSE* (the square root of the *MSE*). It gives prediction errors of different models in the same unit as the variable that is to be predicted. The greater the *r* and the smaller the *RMSE*, the better the network’s performance is considered to be. If several networks had similar results, the one with a smaller hidden layer size was chosen. In accordance with the above-mentioned discrepancies that could occur between training, validation, and the testing of subsets, coefficients of correlation *r*_train_, *r*_val_, and *r*_test_, as well as the values of root mean square error *RMSE*_train_, *RMSE*_val_, and *RMSE*_test_ were also analyzed for the obtained subsets so that balanced prediction of all three microconstituents is ensured, where possible. Results for selected architectures of all three configurations are summarized in Table 5 and Table 6.

The comparison of given metrics overall and for individual subsets shows that these do not differ significantly. That is especially important for training and testing values of coefficients of correlation *r* and root mean square error *RMSE*, i.e., *r*_train_ and *r*_test_, and *RMSE*_train_ and *RMSE*_test_. Based on these indicators, it can be concluded that the design and selection of ANNs ensured robustness and good generalization capabilities.

## 4. Results

When training the ANNs, the error function *MSE* was calculated as a mean for all three outputs. Consequently, *RMSE* values in Table 5 are given as an average for all three outputs. However, it is also useful to see ANNs’ performances for each microstructure constituent individually. Coefficients of correlation *r*_test_, as well as the values of root mean square error *RMSE*_test_ for each microstructure constituent, are given in Table 6. Indices “F-P”, “B” and “M” refer to ferrite–pearlite, bainite, and martensite, respectively.

It can be seen that Configuration No. 1, where all input variables are included, results in the best performance overall, as well as for each microconstituent. Configuration No. 2 including chemical composition along with heat treatment parameters, and Configuration No. 3, which includes specific Jominy distance and heat treatment parameters as inputs, perform similarly, with the latter configuration being marginally better in almost all metrics values. This shows that specific Jominy distance can successfully be used for the prediction of the microstructure of low-alloy steels in situations where the detailed chemical composition of steel is not known. This somewhat aligns with [13], where similar findings were found in the prediction of total hardness after continuous cooling, *HV*_tot_. When the prediction of individual microstructure constituents is observed, it can be seen that all models predict the volume fractions of ferrite–pearlite and martensite better than bainite.

A common practice in the evaluation of ANNs, which also contributes to comparability with results published in the literature, is to use metrics other than coefficients of correlation *r* and the value of root mean square error, *RMSE*, to estimate an ANN’s performance. Various metrics are used for this purpose, such as coefficient of determination, *R*^2^; mean absolute error, *MAE*; mean absolute percentage error, *MAPE*; and others. The *MAPE* value is the most common metric used to measure the accuracy of estimated vs. actual values, i.e., as a forecasting goodness indicator [13]. However, the calculation of *MAPE* implies dividing with target values which are, according to the nature of the problem, sometimes zero, thus making *MAPE* an inadequate metric for assessing an ANN’s performance. Consequently, in this paper, ANNs were additionally evaluated using the coefficient of determination *R*_test_^2^ (Equation (6)) and mean absolute error *MAE* (Equation (7)).

(6) $$R^{2}=1-\frac{\sum_{i}^{n}{\left(t_{i}-y_{i}\right)}^{2}}{\sum_{i}^{n}{\left(t_{i}-\bar{y}\right)}^{2}},$$

(7) $$MAE=\frac{\sum_{i}^{n}\left|\left.t_{i}-y_{i}\right|\right.}{n}.$$

In Equation (6), the numerator represents the residual sum of squares (sum of squared differences between target values *t_i_* and the predicted values *y_i_*), i.e., the variability that the model cannot explain. The denominator represents the total sum of squares (sum of squared differences between target value, *t_i_*, and the mean of the target values, $\bar{y}$), i.e., the total variability in the target values of dependent variables. The coefficient of determination *R*_test_^2^ thus measures how well the predicted values approximate the true data. In Equation (7), the numerator represents the sum of absolute differences between target values *t_i_* and the predicted values *y_i_* for each observation, while *n* in the denominator is the number of observations. Mean absolute error *MAE* measures the average absolute difference between the predicted and target values.

Values of *MAE* are obtained in the same unit as the target variable which enables interpretability, similarly to the *RMSE*. However, *MAE* is more robust regarding outliers since errors are not squared as is the case in the calculation of *RMSE*. Significant differences between *RMSE* and *MAE* values could indicate large error outliers. Table 7 shows *R*_test_^2^ and *MAE*_test_ values for each microstructure constituent. Both metrics show the same trend as *RMSE*_test_ for all three configurations, with Configuration No. 1 being the best (*R*_test_^2^ = 0.887, *MAE*_test_ = 0.084 and *RMSE*_test_ = 0.132) and Configurations No. 2 (*R*_test_^2^ = 0.828, *MAE*_test_ = 0.100 and *RMSE*_test_ = 0.163) and No. 3 (*R*_test_^2^ = 0.820, *MAE*_test_ = 0.102 and *RMSE*_test_ = 0.167) performing slightly worse, but still within acceptable limits.

The trend for individual microconstituents is the same as for *RMSE*_test_ values, i.e., ANNs’ predictions for bainite are somewhat less accurate than for ferrite–pearlite and martensite. For example, the best configuration, No. 1, yields *MAE*_test_ values per microstructure constituent as follows: *MAE*_test,F-P_ = 0.059, *MAE*_test,B_ = 0.105, and *MAE*_test,M_ = 0.087. Although *RMSE* and *MAE* are not to be directly compared, their differences indicate that there are some variances in errors.

## 5. Discussion and Conclusions

In this study, an innovative methodology was developed and applied for predicting the volume fractions of microstructure constituents in heat-treated low-alloy steels using artificial neural networks. The research includes a systematic process for ANN development, starting from the selection of input variables, and going into network training and performance evaluation. By considering three configurations of input variables, the predictive potential of specific Jominy distance as an alternative input when detailed chemical composition is not available was explored. This is advantageous, as it enables practical modeling in scenarios where full material data might be limited.

The use of the “growth method” to limit the ANN’s hidden layer size and the Levenberg–Marquardt algorithm with early stopping to train the networks ensures robust optimization and prevents overfitting. The adoption of the *softmax* transfer function in the output layer, although not typical in the application of ANNs to regression problems, ensures non-negative outputs that sum to 1.

Selection and performance evaluation of ANNs was based on multiple metrics, including root mean square error, *RMSE*, mean absolute error, *MAE*, and coefficient of determination, *R*^2^, ensuring that the networks’ predictive accuracy is thoroughly assessed for both overall and individual microstructure constituents.

Configuration No. 1, which includes all input variables (alloying elements, austenitizing temperature and time, cooling time to 500 °C, and specific Jominy distance), yields the best overall predictive performance. This result was consistent across all metrics, including *RMSE*, *MAE*, and *R*^2^, suggesting that a combination of both chemical composition and specific Jominy distance provides the most accurate representation of microstructure transformations.

Configuration No. 3, in which the chemical composition is replaced with specific Jominy distance as an input variable, performs comparably well. This indicates that specific Jominy distance can effectively replace the chemical composition as an input variable for the prediction of the volume fractions of microstructure constituents. The result aligns well with findings from an earlier study investigating the prediction of total hardness of low-alloy steels after continuous cooling [13], based on Jominy distance as a practical input parameter.

As for individual microstructure constituents, the predictions for ferrite and pearlite, as well as martensite, are more accurate than those for bainite across all configurations. This could be attributed to the complexity of bainite formation and its sensitivity to variations in cooling rates, making it more challenging to model accurately. The research in the field of bainite is extensive [20,21,22,23], and the qualitative theory explaining bainite formation remains somewhat controversial [21,24]. One theory suggests a diffusion-controlled transformation where bainitic growth occurs via a diffusional ledge mechanism, while another proposes that the bainite reaction is a displacive transformation [21]. Both theories have led to the development of models to predict the transformation kinetics [9,10,25].

Overall, this study showed that ANNs are an effective tool for modeling complex, nonlinear relationships relevant in steel microstructure prediction. Once again, it is important to highlight the novelty of this research. The ability to use the specific Jominy distance as a substitute for chemical composition expands the applicability of these models, particularly in industrial settings where detailed material data may not always be available. The research introduces an ANN-based model for predicting steel microstructure using only pre-heat treatment parameters, excluding hardness, which is typically unknown before heat treatment. Additionally, the model can be applied to steels for which experimental data are neither known nor available in the literature, expanding its practical use in process planning and optimization. Industrial implementation of this means that, based on chemical composition and/or specific Jominy distance, heat treatment parameters (by which we can calculate cooling time to 500 °C in every location of a heat-treated part), it is possible to estimate the microstructure of a machine part within seconds with the trained ANN models, which is crucial for its behavior in applications. Future work could focus on improving the prediction of bainite by incorporating additional parameters or exploring alternative network architectures.

## Figures and Tables

**Table 2:** The input variables.

Data No.	Variable	Data No.	Variable
1.	Carbon (C, wt.%)	6.	Nickel (Ni, wt.%)
2.	Silicon (Si, wt.%)	7.	Austenitizing temperature (*T*_a_, °C)
3.	Manganese (Mn, wt.%)	8.	Austenitizing time (*t*_a_, min.)
4.	Chromium (Cr, wt.%)	9.	Cooling time to 500 °C (*t*_500_, s)
5.	Molybdenum (Mo, wt.%)	10.	Specific Jominy distance (*E*_d_, mm)

**Table 3:** Variables used for the development of artificial neural networks.

Variable		Configuration No. 1	Configuration No. 2	Configuration No. 3
Inputs	Carbon (C, wt.%)	**+**	**+**	
	Silicon (Si, wt.%)	**+**	**+**	
	Manganese (Mn, wt.%)	**+**	**+**	
	Chromium (Cr, wt.%)	**+**	**+**	
	Molybdenum (Mo, wt.%)	**+**	**+**	
	Nickel (Ni, wt.%)	**+**	**+**	
	Austenitizing temperature (*T*_a_, °C)	**+**	**+**	**+**
	Austenitizing time (*t*_a_, min.)	**+**	**+**	**+**
	Cooling time to 500 °C (*t*_500_, s)	**+**	**+**	**+**
	Specific Jominy distance (*E*_d_, mm)	**+**		**+**
Outputs	Volume fraction of ferrite–pearlite (*F-P*, -)	**+**	**+**	**+**
	Volume fraction of bainite (*B*, -)	**+**	**+**	**+**
	Volume fraction of martensite (*M*, -)	**+**	**+**	**+**

**Table 4:** Overview of the most important hyperparameters explored for the development of ANNs.

Hyperparameter	Experimented Variables/Values
Number of outputs	1, 3
Normalization of input variables	min–max
Learning algorithm	Levenberg–Marquardt with early stopping
Loss function	mean squared error, *mse*
Number of hidden layers	1
Number of neurons in a hidden layer	determined by the *growth method*
Transfer function (hidden layer)	*tansig*, *logsig*
Transfer functions (output layer)	*linear*, *sigmoid*, *softmax*
Cross-validation	cross-validation (train, val and test subset, random division) *k*-fold cross-validation
Weight initialization	10 iterations

**Table 5:** Performance of selected artificial neural networks.

Config. No.	Hidden Layer Size *H*	Training No.	*r*	*RMSE*	*r*_train_	*RMSE*_train_	*r*_val_	*RMSE*_val_	*r*_test_	*RMSE*_test_
1	5	5	0.932	0.1411	0.941	0.131	0.874	0.187	0.947	0.132
2	9	1	0.922	0.147	0.932	0.138	0.896	0.171	0.905	0.163
3	14	7	0.911	0.158	0.912	0.154	0.902	0.170	0.913	0.167

**Table 6:** Detailed performance information for selected artificial neural networks on test subsets, per microstructure constituent: coefficients of correlation *r*_test_ and root mean square errors *RMSE*_test_.

Config. No.	*r*_test_	*RMSE*_test_	*r*_test,F-P_	*RMSE*_test,F-P_	*r*_test,B_	*RMSE*_test,B_	*r*_test,M_	*RMSE*_test,M_
1	0.963	0.132	0.962	0.113	0.916	0.155	0.963	0.125
2	0.905	0.163	0.931	0.164	0.861	0.165	0.922	0.160
3	0.913	0.167	0.933	0.161	0.868	0.188	0.937	0.148

**Table 7:** Detailed performance of selected artificial neural networks on test dataset, per microstructure constituent: coefficient of determination, *R*_test_^2^, and mean absolute values, *MAE*_test_.

Config. No.	*R*_test_^2^	*MAE*_test_	*MAE*_test,F-P_	*MAE*_test,B_	*MAE*_test,M_
1	0.887	0.084	0.059	0.105	0.087
2	0.828	0.100	0.087	0.105	0.108
3	0.820	0.102	0.083	0.126	0.096

## Data Availability

The original contributions presented in the study are included in the article/Supplementary Material, further inquiries can be directed to the corresponding authors.

## Supplementary Materials

The following supporting information can be downloaded at https://www.mdpi.com/article/10.3390/ma18030564/s1: Table S1: Data used for development and testing of artificial neural networks.

## Nomenclature

Variables	
Al	Aluminum (wt.%)
C	Carbon (wt.%)
Cr	Chromium (wt.%)
Cu	Copper (wt.%)
*E*_d_	Specific Jominy distance (mm)
*H*	Number of neurons in hidden layer, i.e., hidden layer size (-)
*HV*_tot_	Total hardness after continuous cooling (HV)
*I*	Number of input variables (-)
Mn	Manganese (wt.%)
Mo	Molybdenum (wt.%)
*n*	Number of observations (-)
*N*	Total number of datasets/observations (-)
*N*_dof_	Number of degrees of freedom of ANN (-)
Ni	Nickel (wt.%)
*N*_train_	Number of training examples (-)
*N*_traineq_	Number of training equations (-)
*N*_w_	Number of weights in ANN (-)
*O*	Number of output variables (-)
P	Phosphorus (wt.%)
*r*	Coefficient of correlation (-)
*R* ^2^	Coefficient of determination (-)
S	Sulfur (wt.%)
Si	Silicon (wt.%)
*t*_500_	Cooling time to 500 °C (s)
*T*_a_	Austenitizing temperature (°C)
*t*_a_	Austenitizing time (min)
*t*_i_	*i*-th observation target value (-)
V	Vanadium (wt.%)
*y*_i_	*i*-th observation output value (estimated) (-)
$\bar{y}$	Mean value of *y*_i_ (-)
Abbreviations	
*ANN*	Artificial neural network
*MAE*	Mean absolute error
*MSE*	Mean squared error
*RMSE*	Root mean squared error
Subscripts	
B	Bainite
F-P	Ferrite–pearlite
M	Martensite
test	Testing subset
train	Training subset
val	Validation subset

## References

[1] Leitner S., Winter G., Klarner J., Antretter T., Ecker W. (2020). Model-Based Residual Stress Design in Multiphase Seamless Steel Tubes. Materials.

[2] Lopez-Garcia R.D., Medina-Juarez I., Maldonado-Reyes A. (2022). Effect of Quenching Parameters on Distortion Phenomena in AISI 4340 Steel. Metals.

[3] Feng X., Wang Y., Han J., Li Z., Jiang L., Yang B. (2024). Numerical Simulation and Experimental Verification of the Quenching Process for Ti Microalloying H13 Steel Used to Shield Machine Cutter Rings. Metals.

[4] Zener C. (1946). Kinetics of decomposition of austenite. Trans. AIME.

[5] Lusk M.T., Lee Y.-K., Lendvai J., Réti T. (1999). A global material model for simulating the transformation kinetics of low alloy steels. Heat Treatment and Surface Engineering of Light Alloys: Proceedings of the 7th International Seminar of IFHT, Budapest, Hungary, 15–17 September 1999.

[6] Smoljan B., Iljkić D., Smokvina Hanza S., Jokić M., Štic L., Borić A. (2019). Mathematical Modeling and Computer Simulation of Steel Quenching. Mater. Perform. Charact..

[7] Serajzadeh S. (2004). A Mathematical Model for Prediction of Austenite Phase Transformation. Mater. Lett..

[8] Militzer M., Hoyt J.J., Provatas N., Rottler J., Sinclair C.W., Zurob H.S. (2024). Multiscale Modeling of Phase Transformations in Steels. JOM.

[9] Smoljan B., Iljkić D., Smokvina Hanza S., Hajdek K. (2021). Mathematical Modelling of Isothermal Decomposition of Austenite in Steel. Metals.

[10] Quidort D., Brechet Y.J.M. (2002). A model of isothermal and non-isothermal transformation kinetics of bainite in 0.5% C steels. ISIJ Int..

[11] Sitek W., Trzaska J. (2021). Practical Aspects of the Design and Use of the Artificial Neural Networks in Materials Engineering. Metals.

[12] Patel S., Nathani A., Poozesh A., Xu S., Kazempoor P., Ghamarian I. (2024). Combining Neural Networks and Genetic Algorithms to Understand Composition-Microstructure-Property Relationships in Additively Manufactured Metals. J. Manuf. Mater. Process..

[13] Smokvina Hanza S., Marohnić T., Iljkić D., Basan R. (2021). Artificial Neural Networks-Based Prediction of Hardness of Low-Alloy Steels using Specific Jominy Distance. Metals.

[14] Smoljan B., Smokvina Hanza S., Filetin T. Prediction of Phase Transformation Using Neural Networks. Proceedings of the 2nd International Conference Heat Treatment and Surface Engineering in Automotive Applications.

[15] Smokvina Hanza S., Iljkić D., Tomašić N. Modelling of Microstructure Transformation during the steel quenching. Proceedings of the 4th International Ph.D. Conference on Mechanical Engineering.

[16] Liščić B., Totten G.E. (2007). Hardenability. Steel Heat Treatment Handbook: Metallurgy and Technologies.

[17] Rose A., Hougardy H. (1972). Atlas zur Wärmebehandlung der Stähle.

[18] Hagan M.T., Demuth H.B., Beale M.H., De Jesús O. (2014). *Neural Network Design*, 2nd ed. https://hagan.okstate.edu/NNDesign.pdf.

[19] (2022). MATLAB.

[20] Davenport E.S., Bain E.C. (1970). Transformation of austenite at constant subcritical temperatures. Metall. Trans..

[21] Bhadeshia H.K.D.H. (2001). Bainite in Steels: Transformations, Microstructure and Properties.

[22] Fielding L.C.D. (2013). The bainite controversy. Mater. Sci. Technol..

[23] Yang Z.G., Fang H.-S. (2005). An overview on bainite formation in steels. Curr. Opin. Solid State Mater. Sci..

[24] Hillert M. (1995). The nature of bainite. ISIJ Int..

[25] Rees G.I., Bhadeshia H.K.D.H. (1992). Bainite transformation kinetics Part 1 Modified model. Mater. Sci. Technol..

