# Supplementary material for: Possibilities of Using Specific Jominy Distance in ANN Models for Predicting Low-Alloy Steels’ Microstructure
Source: Materials (Basel). 2025 Jan 26;18(3):564. doi: 10.3390/ma18030564 (PMC11818817; doi:10.3390/ma18030564)
Supplement: Supplementary file 1 [file materials-18-00564-s001.zip › materials-3411785-supplementary.pdf]

Table S1. Data used for development and testing of artificial neural networks [17]

Data No.	Steel designation	C *wt.%*	Si *wt.%*	Mn *wt.%*	Cr *wt.%*	Mo *wt.%*	Ni *wt.%*	T_a °C	t_a min	t_{500} s	E_d mm	*F-P* -	*B* -	*M* -
1	Ck15	0.15	0.22	0.41	0.06	0.00	0.06	2.15	900	15	0.71	0.06	0.09	0.85
2	Ck15	0.15	0.22	0.41	0.06	0.00	0.06	2.15	900	15	1.53	0.26	0.22	0.52
3	Ck15	0.15	0.22	0.41	0.06	0.00	0.06	2.15	900	15	2.56	0.65	0.14	0.21
4	Ck15	0.15	0.22	0.41	0.06	0.00	0.06	2.15	900	15	3.86	0.97	0.02	0.01
5	Ck15	0.15	0.22	0.41	0.06	0.00	0.06	2.15	900	15	6.13	1.00	0.00	0.00
6	Ck15	0.15	0.22	0.41	0.06	0.00	0.06	2.15	900	15	18.05	1.00	0.00	0.00
7	Ck15	0.15	0.22	0.41	0.06	0.00	0.06	2.15	900	15	58.93	1.00	0.00	0.00
8	Ck15	0.15	0.22	0.41	0.06	0.00	0.06	2.15	900	15	356.68	1.00	0.00	0.00
9	Ck15	0.15	0.22	0.41	0.06	0.00	0.06	2.15	900	15	2392.82	1.00	0.00	0.00
10	Ck15	0.15	0.22	0.41	0.06	0.00	0.06	2.15	900	15	92288.47	1.00	0.00	0.00
11	Ck15[1]	0.30	0.29	0.39	0.12	0.00	0.00	2.78	930	30	0.71	0.00	0.00	1.00
12	Ck15[1]	0.30	0.29	0.39	0.12	0.00	0.00	2.78	930	30	1.01	0.06	0.03	0.91
13	Ck15[1]	0.30	0.29	0.39	0.12	0.00	0.00	2.78	930	30	1.98	0.14	0.07	0.79
14	Ck15[1]	0.30	0.29	0.39	0.12	0.00	0.00	2.78	930	30	4.06	0.54	0.14	0.32
15	Ck15[1]	0.30	0.29	0.39	0.12	0.00	0.00	2.78	930	30	6.45	0.94	0.02	0.04
16	Ck15[1]	0.30	0.29	0.39	0.12	0.00	0.00	2.78	930	30	13.26	0.98	0.01	0.01
17	Ck15[1]	0.30	0.29	0.39	0.12	0.00	0.00	2.78	930	30	24.58	1.00	0.00	0.00
18	Ck15[1]	0.30	0.29	0.39	0.12	0.00	0.00	2.78	930	30	55.97	1.00	0.00	0.00
19	Ck15[1]	0.30	0.29	0.39	0.12	0.00	0.00	2.78	930	30	261.96	1.00	0.00	0.00
20	Ck15[1]	0.30	0.29	0.39	0.12	0.00	0.00	2.78	930	30	1358.80	1.00	0.00	0.00
21	Ck15[1]	0.30	0.29	0.39	0.12	0.00	0.00	2.78	930	30	79090.32	1.00	0.00	0.00
22	16MnCr5	0.16	0.22	1.12	0.99	0.02	0.12	11.10	870	10	0.64	0.00	0.00	1.00
23	16MnCr5	0.16	0.22	1.12	0.99	0.02	0.12	11.10	870	10	3.47	0.00	0.05	0.95
24	16MnCr5	0.16	0.22	1.12	0.99	0.02	0.12	11.10	870	10	19.91	0.02	0.60	0.38
25	16MnCr5	0.16	0.22	1.12	0.99	0.02	0.12	11.10	870	10	52.87	0.05	0.65	0.30
26	16MnCr5	0.16	0.22	1.12	0.99	0.02	0.12	11.10	870	10	88.40	0.21	0.60	0.19
27	16MnCr5	0.16	0.22	1.12	0.99	0.02	0.12	11.10	870	10	201.17	0.55	0.35	0.10
28	16MnCr5	0.16	0.22	1.12	0.99	0.02	0.12	11.10	870	10	273.84	0.70	0.25	0.05
29	16MnCr5	0.16	0.22	1.12	0.99	0.02	0.12	11.10	870	10	457.84	0.85	0.12	0.03
30	16MnCr5	0.16	0.22	1.12	0.99	0.02	0.12	11.10	870	10	592.00	0.92	0.07	0.01
31	16MnCr5	0.16	0.22	1.12	0.99	0.02	0.12	11.10	870	10	1418.36	1.00	0.00	0.00
32	16MnCr5	0.16	0.22	1.12	0.99	0.02	0.12	11.10	870	10	2371.37	1.00	0.00	0.00
33	16MnCr5	0.16	0.22	1.12	0.99	0.02	0.12	8.33	1050	10	0.60	0.00	0.00	1.00
34	16MnCr5	0.16	0.22	1.12	0.99	0.02	0.12	8.33	1050	10	4.05	0.00	0.10	0.90
35	16MnCr5	0.16	0.22	1.12	0.99	0.02	0.12	8.33	1050	10	18.91	0.00	0.60	0.40
36	16MnCr5	0.16	0.22	1.12	0.99	0.02	0.12	8.33	1050	10	58.59	0.01	0.70	0.29
37	16MnCr5	0.16	0.22	1.12	0.99	0.02	0.12	8.33	1050	10	88.40	0.02	0.70	0.28
38	16MnCr5	0.16	0.22	1.12	0.99	0.02	0.12	8.33	1050	10	155.58	0.05	0.68	0.27
39	16MnCr5	0.16	0.22	1.12	0.99	0.02	0.12	8.33	1050	10	247.09	0.12	0.65	0.23
40	16MnCr5	0.16	0.22	1.12	0.99	0.02	0.12	8.33	1050	10	354.08	0.30	0.55	0.15
41	16MnCr5	0.16	0.22	1.12	0.99	0.02	0.12	8.33	1050	10	848.34	0.52	0.35	0.13
42	16MnCr5	0.16	0.22	1.12	0.99	0.02	0.12	8.33	1050	10	1279.80	0.60	0.35	0.05
43	16MnCr5	0.16	0.22	1.12	0.99	0.02	0.12	8.33	1050	10	6978.31	1.00	0.00	0.00
44	15CrNi6	0.13	0.31	0.51	1.50	0.06	1.55	25.00	870	10	0.71	0.00	0.00	1.00
45	15CrNi6	0.13	0.31	0.51	1.50	0.06	1.55	25.00	870	10	2.74	0.00	0.23	0.77
46	15CrNi6	0.13	0.31	0.51	1.50	0.06	1.55	25.00	870	10	12.30	0.00	0.60	0.40
47	15CrNi6	0.13	0.31	0.51	1.50	0.06	1.55	25.00	870	10	47.22	0.05	0.72	0.23
48	15CrNi6	0.13	0.31	0.51	1.50	0.06	1.55	25.00	870	10	79.23	0.25	0.55	0.20
49	15CrNi6	0.13	0.31	0.51	1.50	0.06	1.55	25.00	870	10	132.92	0.47	0.37	0.16
50	15CrNi6	0.13	0.31	0.51	1.50	0.06	1.55	25.00	870	10	201.08	0.55	0.30	0.15

51	15CrNi6	0.13	0.31	0.51	1.50	0.06	1.55	25.00	870	10	304.19	0.67	0.22	0.11
52	15CrNi6	0.13	0.31	0.51	1.50	0.06	1.55	25.00	870	10	1053.11	0.25	0.09	0.66
53	15CrNi6	0.13	0.31	0.51	1.50	0.06	1.55	25.00	870	10	1677.72	0.37	0.02	0.61
54	15CrNi6	0.13	0.31	0.51	1.50	0.06	1.55	25.00	870	10	7143.85	1.00	0.00	0.00
55	15CrNi6	0.13	0.31	0.51	1.50	0.06	1.55	25.00	870	10	41493.26	1.00	0.00	0.00
56	15CrNi6	0.13	0.31	0.51	1.50	0.06	1.55	25.00	870	10	85621.78	1.00	0.00	0.00
57	15CrNi6	0.13	0.31	0.51	1.50	0.06	1.55	25.56	1050	10	1.21	0.00	0.00	1.00
58	15CrNi6	0.13	0.31	0.51	1.50	0.06	1.55	25.56	1050	10	3.08	0.00	0.05	0.95
59	15CrNi6	0.13	0.31	0.51	1.50	0.06	1.55	25.56	1050	10	13.18	0.00	0.50	0.50
60	15CrNi6	0.13	0.31	0.51	1.50	0.06	1.55	25.56	1050	10	50.86	0.00	0.80	0.20
61	15CrNi6	0.13	0.31	0.51	1.50	0.06	1.55	25.56	1050	10	90.04	0.00	0.83	0.17
62	15CrNi6	0.13	0.31	0.51	1.50	0.06	1.55	25.56	1050	10	159.41	0.01	0.84	0.15
63	15CrNi6	0.13	0.31	0.51	1.50	0.06	1.55	25.56	1050	10	217.69	0.03	0.83	0.14
64	15CrNi6	0.13	0.31	0.51	1.50	0.06	1.55	25.56	1050	10	329.81	0.10	0.77	0.13
65	15CrNi6	0.13	0.31	0.51	1.50	0.06	1.55	25.56	1050	10	1272.47	0.45	0.45	0.10
66	15CrNi6	0.13	0.31	0.51	1.50	0.06	1.55	25.56	1050	10	1566.24	0.50	0.40	0.10
67	15CrNi6	0.13	0.31	0.51	1.50	0.06	1.55	25.56	1050	10	7061.47	0.86	0.02	0.12
68	15CrNi6	0.13	0.31	0.51	1.50	0.06	1.55	25.56	1050	10	41275.93	1.00	0.00	0.00
69	15CrNi6	0.13	0.31	0.51	1.50	0.06	1.55	25.56	1050	10	81074.80	1.00	0.00	0.00
70	20MoCr4[1]	0.28	0.30	0.66	0.56	0.44	0.15	9.72	830	15	0.91	0.00	0.00	1.00
71	20MoCr4[1]	0.28	0.30	0.66	0.56	0.44	0.15	9.72	830	15	4.71	0.00	0.06	0.94
72	20MoCr4[1]	0.28	0.30	0.66	0.56	0.44	0.15	9.72	830	15	19.82	0.00	0.65	0.35
73	20MoCr4[1]	0.28	0.30	0.66	0.56	0.44	0.15	9.72	830	15	64.56	0.00	0.86	0.14
74	20MoCr4[1]	0.28	0.30	0.66	0.56	0.44	0.15	9.72	830	15	221.43	0.15	0.75	0.10
75	20MoCr4[1]	0.28	0.30	0.66	0.56	0.44	0.15	9.72	830	15	454.41	0.43	0.50	0.07
76	20MoCr4[1]	0.28	0.30	0.66	0.56	0.44	0.15	9.72	830	15	1145.19	0.60	0.36	0.04
77	20MoCr4[1]	0.28	0.30	0.66	0.56	0.44	0.15	9.72	830	15	2350.16	0.70	0.27	0.03
78	20MoCr4[1]	0.28	0.30	0.66	0.56	0.44	0.15	9.72	830	15	6909.22	1.00	0.00	0.00
79	20MoCr4[1]	0.28	0.30	0.66	0.56	0.44	0.15	9.72	830	15	81264.69	1.00	0.00	0.00
80	20MoCr4[1]	0.28	0.30	0.66	0.56	0.44	0.15	10.41	930	30	0.57	0.00	0.00	1.00
81	20MoCr4[1]	0.28	0.30	0.66	0.56	0.44	0.15	10.41	930	30	1.60	0.00	0.00	1.00
82	20MoCr4[1]	0.28	0.30	0.66	0.56	0.44	0.15	10.41	930	30	3.64	0.00	0.01	0.99
83	20MoCr4[1]	0.28	0.30	0.66	0.56	0.44	0.15	10.41	930	30	7.10	0.00	0.10	0.90
84	20MoCr4[1]	0.28	0.30	0.66	0.56	0.44	0.15	10.41	930	30	25.62	0.00	0.69	0.31
85	20MoCr4[1]	0.28	0.30	0.66	0.56	0.44	0.15	10.41	930	30	79.29	0.00	0.85	0.15
86	20MoCr4[1]	0.28	0.30	0.66	0.56	0.44	0.15	10.41	930	30	245.38	0.14	0.77	0.09
87	20MoCr4[1]	0.28	0.30	0.66	0.56	0.44	0.15	10.41	930	30	410.06	0.34	0.58	0.08
88	20MoCr4[1]	0.28	0.30	0.66	0.56	0.44	0.15	10.41	930	30	1406.31	0.55	0.40	0.05
89	20MoCr4[1]	0.28	0.30	0.66	0.56	0.44	0.15	10.41	930	30	3198.21	0.65	0.31	0.04
90	20MoCr4[1]	0.28	0.30	0.66	0.56	0.44	0.15	10.41	930	30	9897.82	0.97	0.02	0.01
91	20MoCr4[1]	0.28	0.30	0.66	0.56	0.44	0.15	10.41	930	30	18329.81	1.00	0.00	0.00
92	20MoCr4[1]	0.28	0.30	0.66	0.56	0.44	0.15	10.41	930	30	116415.95	1.00	0.00	0.00
93	20MoCr4[1]	0.57	0.30	0.66	0.56	0.44	0.15	29.16	830	15	0.40	0.00	0.00	1.00
94	20MoCr4[1]	0.57	0.30	0.66	0.56	0.44	0.15	29.16	830	15	3.12	0.00	0.00	1.00
95	20MoCr4[1]	0.57	0.30	0.66	0.56	0.44	0.15	29.16	830	15	16.99	0.00	0.00	1.00
96	20MoCr4[1]	0.57	0.30	0.66	0.56	0.44	0.15	29.16	830	15	49.94	0.00	0.07	0.93
97	20MoCr4[1]	0.57	0.30	0.66	0.56	0.44	0.15	29.16	830	15	139.48	0.00	0.14	0.86
98	20MoCr4[1]	0.57	0.30	0.66	0.56	0.44	0.15	29.16	830	15	721.38	0.00	0.95	0.05
99	20MoCr4[1]	0.57	0.30	0.66	0.56	0.44	0.15	29.16	830	15	1205.53	0.01	0.98	0.01
100	20MoCr4[1]	0.57	0.30	0.66	0.56	0.44	0.15	29.16	830	15	2473.99	1.00	0.00	0.00
101	20MoCr4[1]	0.57	0.30	0.66	0.56	0.44	0.15	29.16	830	15	6909.22	1.00	0.00	0.00
102	20MoCr4[1]	0.57	0.30	0.66	0.56	0.44	0.15	29.16	830	15	12795.21	1.00	0.00	0.00
103	20MoCr4[1]	0.57	0.30	0.66	0.56	0.44	0.15	29.15	830	15	27642.04	1.00	0.00	0.00
104	20MoCr4[1]	0.57	0.30	0.66	0.56	0.44	0.15	29.16	830	15	85546.73	1.00	0.00	0.00

105	20MoCr4[1]	0.57	0.30	0.66	0.56	0.44	0.15	27.08	930	30	0.86	0.00	0.00	1.00
106	20MoCr4[1]	0.57	0.30	0.66	0.56	0.44	0.15	27.08	930	30	16.14	0.00	0.00	1.00
107	20MoCr4[1]	0.57	0.30	0.66	0.56	0.44	0.15	27.08	930	30	52.58	0.00	0.06	0.94
108	20MoCr4[1]	0.57	0.30	0.66	0.56	0.44	0.15	27.08	930	30	146.83	0.00	0.13	0.87
109	20MoCr4[1]	0.57	0.30	0.66	0.56	0.44	0.15	27.08	930	30	807.65	0.00	0.95	0.05
110	20MoCr4[1]	0.57	0.30	0.66	0.56	0.44	0.15	27.08	930	30	1406.31	0.01	0.97	0.02
111	20MoCr4[1]	0.57	0.30	0.66	0.56	0.44	0.15	27.08	930	30	2741.59	0.03	0.97	0.00
112	20MoCr4[1]	0.57	0.30	0.66	0.56	0.44	0.15	27.08	930	30	8484.67	0.10	0.90	0.00
113	20MoCr4[1]	0.57	0.30	0.66	0.56	0.44	0.15	27.08	930	30	30631.84	1.00	0.00	0.00
114	20MoCr4[1]	0.57	0.30	0.66	0.56	0.44	0.15	27.08	930	30	105053.24	1.00	0.00	0.00
115	25MoCr4	0.31	0.20	0.67	0.50	0.45	0.11	13.89	830	15	0.74	0.00	0.00	1.00
116	25MoCr4	0.31	0.20	0.67	0.50	0.45	0.11	13.89	830	15	3.13	0.00	0.01	0.99
117	25MoCr4	0.31	0.20	0.67	0.50	0.45	0.11	13.89	830	15	7.50	0.00	0.16	0.84
118	25MoCr4	0.31	0.20	0.67	0.50	0.45	0.11	13.89	830	15	23.23	0.00	0.74	0.26
119	25MoCr4	0.31	0.20	0.67	0.50	0.45	0.11	13.89	830	15	64.94	0.00	0.82	0.18
120	25MoCr4	0.31	0.20	0.67	0.50	0.45	0.11	13.89	830	15	211.78	0.50	0.38	0.12
121	25MoCr4	0.31	0.20	0.67	0.50	0.45	0.11	13.89	830	15	434.90	0.61	0.30	0.09
122	25MoCr4	0.31	0.20	0.67	0.50	0.45	0.11	13.89	830	15	1215.69	0.69	0.25	0.06
123	25MoCr4	0.31	0.20	0.67	0.50	0.45	0.11	13.89	830	15	2628.11	0.82	0.15	0.03
124	25MoCr4	0.31	0.20	0.67	0.50	0.45	0.11	13.89	830	15	6978.31	1.00	0.00	0.00
125	25MoCr4	0.31	0.20	0.67	0.50	0.45	0.11	13.89	830	15	86596.43	1.00	0.00	0.00
126	25MoCr4	0.31	0.20	0.67	0.50	0.45	0.11	19.44	930	30	0.66	0.00	0.00	1.00
127	25MoCr4	0.31	0.20	0.67	0.50	0.45	0.11	19.44	930	30	4.19	0.00	0.02	0.98
128	25MoCr4	0.31	0.20	0.67	0.50	0.45	0.11	19.44	930	30	9.50	0.00	0.20	0.80
129	25MoCr4	0.31	0.20	0.67	0.50	0.45	0.11	19.44	930	30	27.83	0.00	0.74	0.26
130	25MoCr4	0.31	0.20	0.67	0.50	0.45	0.11	19.44	930	30	77.43	0.00	0.83	0.17
131	25MoCr4	0.31	0.20	0.67	0.50	0.45	0.11	19.44	930	30	110.78	0.03	0.82	0.15
132	25MoCr4	0.31	0.20	0.67	0.50	0.45	0.11	19.44	930	30	238.66	0.31	0.57	0.12
133	25MoCr4	0.31	0.20	0.67	0.50	0.45	0.11	19.44	930	30	488.53	0.54	0.37	0.09
134	25MoCr4	0.31	0.20	0.67	0.50	0.45	0.11	19.44	930	30	1359.36	0.65	0.31	0.04
135	25MoCr4	0.31	0.20	0.67	0.50	0.45	0.11	19.44	930	30	3082.40	0.80	0.20	0.00
136	25MoCr4	0.31	0.20	0.67	0.50	0.45	0.11	19.44	930	30	18478.50	1.00	0.00	0.00
137	25MoCr4	0.31	0.20	0.67	0.50	0.45	0.11	19.44	930	30	110775.69	1.00	0.00	0.00
138	20NiMoCr6[1]	0.28	0.15	0.62	0.47	0.48	1.58	36.11	930	30	0.60	0.00	0.00	1.00
139	20NiMoCr6[1]	0.28	0.15	0.62	0.47	0.48	1.58	36.11	930	30	2.93	0.00	0.00	1.00
140	20NiMoCr6[1]	0.28	0.15	0.62	0.47	0.48	1.58	36.11	930	30	23.87	0.00	0.03	0.97
141	20NiMoCr6[1]	0.28	0.15	0.62	0.47	0.48	1.58	36.11	930	30	59.95	0.00	0.50	0.50
142	20NiMoCr6[1]	0.28	0.15	0.62	0.47	0.48	1.58	36.11	930	30	251.19	0.00	0.87	0.13
143	20NiMoCr6[1]	0.28	0.15	0.62	0.47	0.48	1.58	36.11	930	30	814.91	0.00	0.90	0.10
144	20NiMoCr6[1]	0.28	0.15	0.62	0.47	0.48	1.58	36.11	930	30	3082.40	0.01	0.92	0.07
145	20NiMoCr6[1]	0.28	0.15	0.62	0.47	0.48	1.58	36.11	930	30	18478.50	0.26	0.70	0.04
146	20NiMoCr6[1]	0.28	0.15	0.62	0.47	0.48	1.58	36.11	930	30	48852.74	0.55	0.43	0.02
147	20NiMoCr6[1]	0.28	0.15	0.62	0.47	0.48	1.58	36.11	930	30	100000.00	1.00	0.00	0.00
148	Ck45	0.44	0.22	0.66	0.15	0.00	0.00	5	880	3	1.8	0.00	0.02	0.98
149	Ck45	0.44	0.22	0.66	0.15	0.00	0.00	5	880	3	2.8	0.11	0.20	0.69
150	Ck45	0.44	0.22	0.66	0.15	0.00	0.00	5	880	3	5.3	0.13	0.17	0.70
151	Ck45	0.44	0.22	0.66	0.15	0.00	0.00	5	880	3	8.8	0.90	0.05	0.05
152	Ck45	0.44	0.22	0.66	0.15	0.00	0.00	5	880	3	17.8	1.00	0.00	0.00
153	Ck45	0.44	0.22	0.66	0.15	0.00	0.00	5	880	3	73.3	1.00	0.00	0.00
154	Ck45	0.44	0.22	0.66	0.15	0.00	0.00	5	880	3	714.4	1.00	0.00	0.00
155	Ck45	0.44	0.22	0.66	0.15	0.00	0.00	5	880	3	32874.1	1.00	0.00	0.00
156	Ck45	0.44	0.22	0.66	0.15	0.00	0.00	5	1050	3	2.2	0.00	0.01	0.99
157	Ck45	0.44	0.22	0.66	0.15	0.00	0.00	5	1050	3	3.5	0.00	0.02	0.98
158	Ck45	0.44	0.22	0.66	0.15	0.00	0.00	5	1050	3	5.0	0.03	0.03	0.94

159	Ck45	0.44	0.22	0.66	0.15	0.00	0.00	5	1050	3	8.3	0.11	0.20	0.69
160	Ck45	0.44	0.22	0.66	0.15	0.00	0.00	5	1050	3	14.0	0.33	0.40	0.27
161	Ck45	0.44	0.22	0.66	0.15	0.00	0.00	5	1050	3	51.0	1.00	0.00	0.00
162	Ck45	0.44	0.22	0.66	0.15	0.00	0.00	5	1050	3	136.4	1.00	0.00	0.00
163	Ck45	0.44	0.22	0.66	0.15	0.00	0.00	5	1050	3	925.3	1.00	0.00	0.00
164	Ck45	0.44	0.22	0.66	0.15	0.00	0.00	5	1050	3	32874.1	1.00	0.00	0.00
165	37MnSi5	0.38	1.05	1.14	0.23	0.00	0.00	9	860	7	2.9	0.00	0.01	0.99
166	37MnSi5	0.38	1.05	1.14	0.23	0.00	0.00	9	860	7	6.6	0.01	0.10	0.89
167	37MnSi5	0.38	1.05	1.14	0.23	0.00	0.00	9	860	7	13.6	0.02	0.30	0.68
168	37MnSi5	0.38	1.05	1.14	0.23	0.00	0.00	9	860	7	30.8	0.35	0.30	0.35
169	37MnSi5	0.38	1.05	1.14	0.23	0.00	0.00	9	860	7	51.4	0.90	0.05	0.05
170	37MnSi5	0.38	1.05	1.14	0.23	0.00	0.00	9	860	7	69.9	1.00	0.00	0.00
171	37MnSi5	0.38	1.05	1.14	0.23	0.00	0.00	9	860	7	135.9	1.00	0.00	0.00
172	37MnSi5	0.38	1.05	1.14	0.23	0.00	0.00	9	860	7	292.9	1.00	0.00	0.00
173	37MnSi5	0.38	1.05	1.14	0.23	0.00	0.00	9	860	7	1107.8	1.00	0.00	0.00
174	37MnSi5	0.38	1.05	1.14	0.23	0.00	0.00	9	860	7	7356.4	1.00	0.00	0.00
175	37MnSi5	0.38	1.05	1.14	0.23	0.00	0.00	15	1050	5	3.1	0.00	0.01	0.99
176	37MnSi5	0.38	1.05	1.14	0.23	0.00	0.00	15	1050	5	7.0	0.00	0.03	0.97
177	37MnSi5	0.38	1.05	1.14	0.23	0.00	0.00	15	1050	5	13.6	0.01	0.10	0.89
178	37MnSi5	0.38	1.05	1.14	0.23	0.00	0.00	15	1050	5	26.4	0.02	0.40	0.58
179	37MnSi5	0.38	1.05	1.14	0.23	0.00	0.00	15	1050	5	69.9	0.60	0.15	0.25
180	37MnSi5	0.38	1.05	1.14	0.23	0.00	0.00	15	1050	5	135.9	1.00	0.00	0.00
181	37MnSi5	0.38	1.05	1.14	0.23	0.00	0.00	15	1050	5	324.4	1.00	0.00	0.00
182	37MnSi5	0.38	1.05	1.14	0.23	0.00	0.00	15	1050	5	1052.5	1.00	0.00	0.00
183	37MnSi5	0.38	1.05	1.14	0.23	0.00	0.00	15	1050	5	7742.6	1.00	0.00	0.00
184	42MnV7	0.43	0.28	1.67	0.32	0.03	0.11	16	870	8	4.7	0.00	0.00	1.00
185	42MnV7	0.43	0.28	1.67	0.32	0.03	0.11	16	870	8	10.3	0.00	0.02	0.98
186	42MnV7	0.43	0.28	1.67	0.32	0.03	0.11	16	870	8	43.7	0.00	0.75	0.25
187	42MnV7	0.43	0.28	1.67	0.32	0.03	0.11	16	870	8	77.2	0.02	0.75	0.23
188	42MnV7	0.43	0.28	1.67	0.32	0.03	0.11	16	870	8	129.5	0.08	0.80	0.12
189	42MnV7	0.43	0.28	1.67	0.32	0.03	0.11	16	870	8	296.4	0.70	0.20	0.10
190	42MnV7	0.43	0.28	1.67	0.32	0.03	0.11	16	870	8	1026.2	1.00	0.00	0.00
191	42MnV7	0.43	0.28	1.67	0.32	0.03	0.11	16	870	8	6961.4	1.00	0.00	0.00
192	42MnV7	0.43	0.28	1.67	0.32	0.03	0.11	16	870	8	79227.6	1.00	0.00	0.00
193	42MnV7	0.43	0.28	1.67	0.32	0.03	0.11	31	1050	8	8.8	0.00	0.00	1.00
194	42MnV7	0.43	0.28	1.67	0.32	0.03	0.11	31	1050	8	20.1	0.00	0.05	0.95
195	42MnV7	0.43	0.28	1.67	0.32	0.03	0.11	31	1050	8	39.4	0.00	0.15	0.85
196	42MnV7	0.43	0.28	1.67	0.32	0.03	0.11	31	1050	8	136.4	0.04	0.80	0.16
197	42MnV7	0.43	0.28	1.67	0.32	0.03	0.11	31	1050	8	328.7	0.20	0.75	0.05
198	42MnV7	0.43	0.28	1.67	0.32	0.03	0.11	31	1050	8	1026.2	0.98	0.01	0.01
199	42MnV7	0.43	0.28	1.67	0.32	0.03	0.11	31	1050	8	6277.0	1.00	0.00	0.00
200	42MnV7	0.43	0.28	1.67	0.32	0.03	0.11	31	1050	8	47223.4	1.00	0.00	0.00
201	42MnV7	0.43	0.28	1.67	0.32	0.03	0.11	31	1050	8	79227.6	1.00	0.00	0.00
202	34Cr4	0.35	0.23	0.65	1.11	0.05	0.23	17	850	8	6.8	0.00	0.00	1.00
203	34Cr4	0.35	0.23	0.65	1.11	0.05	0.23	17	850	8	12.6	0.00	0.04	0.96
204	34Cr4	0.35	0.23	0.65	1.11	0.05	0.23	17	850	8	23.5	0.00	0.30	0.70
205	34Cr4	0.35	0.23	0.65	1.11	0.05	0.23	17	850	8	48.5	0.00	0.60	0.40
206	34Cr4	0.35	0.23	0.65	1.11	0.05	0.23	17	850	8	77.2	0.01	0.75	0.24
207	34Cr4	0.35	0.23	0.65	1.11	0.05	0.23	17	850	8	136.4	0.13	0.70	0.17
208	34Cr4	0.35	0.23	0.65	1.11	0.05	0.23	17	850	8	228.8	0.70	0.15	0.15
209	34Cr4	0.35	0.23	0.65	1.11	0.05	0.23	17	850	8	312.2	1.00	0.00	0.00
210	34Cr4	0.35	0.23	0.65	1.11	0.05	0.23	17	850	8	678.4	1.00	0.00	0.00
211	34Cr4	0.35	0.23	0.65	1.11	0.05	0.23	17	850	8	6610.4	1.00	0.00	0.00
212	34Cr4	0.35	0.23	0.65	1.11	0.05	0.23	17	850	8	83435.0	1.00	0.00	0.00

213	34Cr4	0.35	0.23	0.65	1.11	0.05	0.23	17	1050	8	6.8	0.00	0.00	1.00
214	34Cr4	0.35	0.23	0.65	1.11	0.05	0.23	17	1050	8	10.8	0.00	0.01	0.99
215	34Cr4	0.35	0.23	0.65	1.11	0.05	0.23	17	1050	8	21.2	0.00	0.30	0.70
216	34Cr4	0.35	0.23	0.65	1.11	0.05	0.23	17	1050	8	77.2	0.00	0.75	0.25
217	34Cr4	0.35	0.23	0.65	1.11	0.05	0.23	17	1050	8	217.3	0.01	0.95	0.04
218	34Cr4	0.35	0.23	0.65	1.11	0.05	0.23	17	1050	8	425.8	0.94	0.05	0.01
219	34Cr4	0.35	0.23	0.65	1.11	0.05	0.23	17	1050	8	611.7	0.97	0.02	0.01
220	34Cr4	0.35	0.23	0.65	1.11	0.05	0.23	17	1050	8	974.5	1.00	0.00	0.00
221	34Cr4	0.35	0.23	0.65	1.11	0.05	0.23	17	1050	8	6961.4	1.00	0.00	0.00
222	34Cr4	0.35	0.23	0.65	1.11	0.05	0.23	17	1050	8	71438.5	1.00	0.00	0.00
223	34Cr4	0.36	0.29	0.69	1.09	0.07	0.08	13	850	8	2.7	0.00	0.00	1.00
224	34Cr4	0.36	0.29	0.69	1.09	0.07	0.08	13	850	8	6.8	0.00	0.03	0.97
225	34Cr4	0.36	0.29	0.69	1.09	0.07	0.08	13	850	8	15.5	0.00	0.15	0.85
226	34Cr4	0.36	0.29	0.69	1.09	0.07	0.08	13	850	8	24.7	0.01	0.40	0.59
227	34Cr4	0.36	0.29	0.69	1.09	0.07	0.08	13	850	8	48.5	0.04	0.75	0.21
228	34Cr4	0.36	0.29	0.69	1.09	0.07	0.08	13	850	8	81.3	0.42	0.30	0.28
229	34Cr4	0.36	0.29	0.69	1.09	0.07	0.08	13	850	8	136.4	1.00	0.00	0.00
230	34Cr4	0.36	0.29	0.69	1.09	0.07	0.08	13	850	8	296.4	1.00	0.00	0.00
231	34Cr4	0.36	0.29	0.69	1.09	0.07	0.08	13	850	8	1138.1	1.00	0.00	0.00
232	41Cr4	0.44	0.22	0.80	1.04	0.04	0.26	23	840	8	7.1	0.00	0.00	1.00
233	41Cr4	0.44	0.22	0.80	1.04	0.04	0.26	23	840	8	10.8	0.00	0.03	0.97
234	41Cr4	0.44	0.22	0.80	1.04	0.04	0.26	23	840	8	23.5	0.00	0.05	0.95
235	41Cr4	0.44	0.22	0.80	1.04	0.04	0.26	23	840	8	41.5	0.00	0.60	0.40
236	41Cr4	0.44	0.22	0.80	1.04	0.04	0.26	23	840	8	62.8	0.02	0.70	0.28
237	41Cr4	0.44	0.22	0.80	1.04	0.04	0.26	23	840	8	129.5	0.07	0.60	0.33
238	41Cr4	0.44	0.22	0.80	1.04	0.04	0.26	23	840	8	206.4	0.56	0.25	0.19
239	41Cr4	0.44	0.22	0.80	1.04	0.04	0.26	23	840	8	296.4	1.00	0.00	0.00
240	41Cr4	0.44	0.22	0.80	1.04	0.04	0.26	23	840	8	644.2	1.00	0.00	0.00
241	41Cr4	0.44	0.22	0.80	1.04	0.04	0.26	23	840	8	6277.0	1.00	0.00	0.00
242	41Cr4	0.44	0.22	0.80	1.04	0.04	0.26	23	840	8	80051.7	1.00	0.00	0.00
243	41Cr4	0.44	0.22	0.80	1.04	0.04	0.26	24	1050	8	5.7	0.00	0.00	1.00
244	41Cr4	0.44	0.22	0.80	1.04	0.04	0.26	24	1050	8	11.4	0.00	0.01	0.99
245	41Cr4	0.44	0.22	0.80	1.04	0.04	0.26	24	1050	8	41.5	0.00	0.50	0.50
246	41Cr4	0.44	0.22	0.80	1.04	0.04	0.26	24	1050	8	136.4	0.02	0.65	0.33
247	41Cr4	0.44	0.22	0.80	1.04	0.04	0.26	24	1050	8	312.2	0.08	0.70	0.22
248	41Cr4	0.44	0.22	0.80	1.04	0.04	0.26	24	1050	8	644.2	1.00	0.00	0.00
249	41Cr4	0.44	0.22	0.80	1.04	0.04	0.26	24	1050	8	6610.4	1.00	0.00	0.00
250	41Cr4	0.44	0.22	0.80	1.04	0.04	0.26	24	1050	8	80051.7	1.00	0.00	0.00
251	41Cr4	0.41	0.25	0.71	1.06	0.02	0.22	21	840	8	6.1	0.00	0.00	1.00
252	41Cr4	0.41	0.25	0.71	1.06	0.02	0.22	21	840	8	11.4	0.00	0.02	0.98
253	41Cr4	0.41	0.25	0.71	1.06	0.02	0.22	21	840	8	21.2	0.00	0.05	0.95
254	41Cr4	0.41	0.25	0.71	1.06	0.02	0.22	21	840	8	48.5	0.00	0.60	0.40
255	41Cr4	0.41	0.25	0.71	1.06	0.02	0.22	21	840	8	69.6	0.02	0.60	0.38
256	41Cr4	0.41	0.25	0.71	1.06	0.02	0.22	21	840	8	136.4	0.55	0.25	0.20
257	41Cr4	0.41	0.25	0.71	1.06	0.02	0.22	21	840	8	217.3	1.00	0.00	0.00
258	41Cr4	0.41	0.25	0.71	1.06	0.02	0.22	21	840	8	312.2	1.00	0.00	0.00
259	41Cr4	0.41	0.25	0.71	1.06	0.02	0.22	21	840	8	1138.1	1.00	0.00	0.00
260	36Cr6	0.36	0.25	0.49	1.54	0.03	0.21	24	860	10	9.5	0.00	0.00	1.00
261	36Cr6	0.36	0.25	0.49	1.54	0.03	0.21	24	860	10	15.5	0.00	0.03	0.97
262	36Cr6	0.36	0.25	0.49	1.54	0.03	0.21	24	860	10	43.7	0.00	0.50	0.50
263	36Cr6	0.36	0.25	0.49	1.54	0.03	0.21	24	860	10	136.4	0.02	0.80	0.18
264	36Cr6	0.36	0.25	0.49	1.54	0.03	0.21	24	860	10	312.2	0.17	0.80	0.03
265	36Cr6	0.36	0.25	0.49	1.54	0.03	0.21	24	860	10	678.4	1.00	0.00	0.00
266	36Cr6	0.36	0.25	0.49	1.54	0.03	0.21	24	860	10	2742.9	1.00	0.00	0.00

267	36Cr6	0.36	0.25	0.49	1.54	0.03	0.21	24	860	10	75232.3	1.00	0.00	0.00
268	36Cr6	0.36	0.25	0.49	1.54	0.03	0.21	26	1050	10	15.5	0.00	0.00	1.00
269	36Cr6	0.36	0.25	0.49	1.54	0.03	0.21	26	1050	10	43.7	0.00	0.65	0.35
270	36Cr6	0.36	0.25	0.49	1.54	0.03	0.21	26	1050	10	136.4	0.00	0.95	0.05
271	36Cr6	0.36	0.25	0.49	1.54	0.03	0.21	26	1050	10	346.2	0.01	0.95	0.04
272	36Cr6	0.36	0.25	0.49	1.54	0.03	0.21	26	1050	10	678.4	0.88	0.11	0.01
273	36Cr6	0.36	0.25	0.49	1.54	0.03	0.21	26	1050	10	1138.1	1.00	0.00	0.00
274	36Cr6	0.36	0.25	0.49	1.54	0.03	0.21	26	1050	10	5960.5	1.00	0.00	0.00
275	36Cr6	0.36	0.25	0.49	1.54	0.03	0.21	26	1050	10	75232.3	1.00	0.00	0.00
276	25CrMo4	0.22	0.25	0.64	0.97	0.23	0.33	14	875	10	3.5	0.00	0.05	0.95
277	25CrMo4	0.22	0.25	0.64	0.97	0.23	0.33	14	875	10	7.9	0.00	0.05	0.95
278	25CrMo4	0.22	0.25	0.64	0.97	0.23	0.33	14	875	10	14.0	0.00	0.25	0.75
279	25CrMo4	0.22	0.25	0.64	0.97	0.23	0.33	14	875	10	46.0	0.01	0.40	0.59
280	25CrMo4	0.22	0.25	0.64	0.97	0.23	0.33	14	875	10	66.1	0.02	0.60	0.38
281	25CrMo4	0.22	0.25	0.64	0.97	0.23	0.33	14	875	10	123.0	0.03	0.75	0.22
282	25CrMo4	0.22	0.25	0.64	0.97	0.23	0.33	14	875	10	346.2	0.35	0.60	0.05
283	25CrMo4	0.22	0.25	0.64	0.97	0.23	0.33	14	875	10	800.5	0.65	0.30	0.05
284	25CrMo4	0.22	0.25	0.64	0.97	0.23	0.33	14	875	10	1198.5	1.00	0.00	0.00
285	25CrMo4	0.22	0.25	0.64	0.97	0.23	0.33	14	875	10	2348.5	1.00	0.00	0.00
286	25CrMo4	0.22	0.25	0.64	0.97	0.23	0.33	14	875	10	8562.2	1.00	0.00	0.00
287	25CrMo4	0.22	0.25	0.64	0.97	0.23	0.33	14	875	10	80051.7	1.00	0.00	0.00
288	25CrMo4	0.22	0.25	0.64	0.97	0.23	0.33	17	1050	10	1.5	0.00	0.01	0.99
289	25CrMo4	0.22	0.25	0.64	0.97	0.23	0.33	17	1050	10	3.3	0.00	0.02	0.98
290	25CrMo4	0.22	0.25	0.64	0.97	0.23	0.33	17	1050	10	14.7	0.00	0.30	0.70
291	25CrMo4	0.22	0.25	0.64	0.97	0.23	0.33	17	1050	10	105.3	0.00	0.70	0.30
292	25CrMo4	0.22	0.25	0.64	0.97	0.23	0.33	17	1050	10	383.9	0.02	0.97	0.01
293	25CrMo4	0.22	0.25	0.64	0.97	0.23	0.33	17	1050	10	792.3	0.05	0.94	0.01
294	25CrMo4	0.22	0.25	0.64	0.97	0.23	0.33	17	1050	10	1262.2	0.20	0.79	0.01
295	25CrMo4	0.22	0.25	0.64	0.97	0.23	0.33	17	1050	10	2473.2	0.35	0.64	0.01
296	25CrMo4	0.22	0.25	0.64	0.97	0.23	0.33	17	1050	10	4461.1	0.95	0.04	0.01
297	25CrMo4	0.22	0.25	0.64	0.97	0.23	0.33	17	1050	10	9016.9	1.00	0.00	0.00
298	25CrMo4	0.22	0.25	0.64	0.97	0.23	0.33	17	1050	10	71438.5	1.00	0.00	0.00
299	34CrMo4	0.30	0.22	0.64	1.01	0.24	0.11	16	850	10	3.1	0.00	0.02	0.98
300	34CrMo4	0.30	0.22	0.64	1.01	0.24	0.11	16	850	10	13.3	0.00	0.50	0.50
301	34CrMo4	0.30	0.22	0.64	1.01	0.24	0.11	16	850	10	43.7	0.05	0.60	0.35
302	34CrMo4	0.30	0.22	0.64	1.01	0.24	0.11	16	850	10	77.2	0.08	0.70	0.22
303	34CrMo4	0.30	0.22	0.64	1.01	0.24	0.11	16	850	10	136.4	0.10	0.80	0.10
304	34CrMo4	0.30	0.22	0.64	1.01	0.24	0.11	16	850	10	328.7	0.30	0.68	0.02
305	34CrMo4	0.30	0.22	0.64	1.01	0.24	0.11	16	850	10	714.4	0.69	0.30	0.01
306	34CrMo4	0.30	0.22	0.64	1.01	0.24	0.11	16	850	10	1138.1	1.00	0.00	0.00
307	34CrMo4	0.30	0.22	0.64	1.01	0.24	0.11	16	850	10	8130.4	1.00	0.00	0.00
308	34CrMo4	0.30	0.22	0.64	1.01	0.24	0.11	16	850	10	75232.3	1.00	0.00	0.00
309	34CrMo4	0.30	0.22	0.64	1.01	0.24	0.11	20	1050	10	3.0	0.00	0.00	1.00
310	34CrMo4	0.30	0.22	0.64	1.01	0.24	0.11	20	1050	10	8.3	0.00	0.10	0.90
311	34CrMo4	0.30	0.22	0.64	1.01	0.24	0.11	20	1050	10	39.4	0.00	0.70	0.30
312	34CrMo4	0.30	0.22	0.64	1.01	0.24	0.11	20	1050	10	85.6	0.00	0.80	0.20
313	34CrMo4	0.30	0.22	0.64	1.01	0.24	0.11	20	1050	10	186.1	0.00	0.95	0.05
314	34CrMo4	0.30	0.22	0.64	1.01	0.24	0.11	20	1050	10	346.2	0.02	0.95	0.03
315	34CrMo4	0.30	0.22	0.64	1.01	0.24	0.11	20	1050	10	714.4	0.05	0.92	0.03
316	34CrMo4	0.30	0.22	0.64	1.01	0.24	0.11	20	1050	10	2230.1	0.20	0.79	0.01
317	34CrMo4	0.30	0.22	0.64	1.01	0.24	0.11	20	1050	10	6961.4	1.00	0.00	0.00
318	34CrMo4	0.30	0.22	0.64	1.01	0.24	0.11	20	1050	10	71438.5	1.00	0.00	0.00
319	42CrMo4	0.38	0.23	0.64	0.99	0.16	0.08	18	850	10	3.1	0.00	0.00	1.00
320	42CrMo4	0.38	0.23	0.64	0.99	0.16	0.08	18	850	10	6.0	0.00	0.02	0.98

321	42CrMo4	0.38	0.23	0.64	0.99	0.16	0.08	18	850	10	14.0	0.00	0.03	0.97
322	42CrMo4	0.38	0.23	0.64	0.99	0.16	0.08	18	850	10	43.7	0.02	0.75	0.23
323	42CrMo4	0.38	0.23	0.64	0.99	0.16	0.08	18	850	10	206.4	0.12	0.85	0.03
324	42CrMo4	0.38	0.23	0.64	0.99	0.16	0.08	18	850	10	328.7	0.22	0.75	0.03
325	42CrMo4	0.38	0.23	0.64	0.99	0.16	0.08	18	850	10	974.5	1.00	0.00	0.00
326	42CrMo4	0.38	0.23	0.64	0.99	0.16	0.08	18	850	10	6961.4	1.00	0.00	0.00
327	42CrMo4	0.38	0.23	0.64	0.99	0.16	0.08	18	850	10	67836.1	1.00	0.00	0.00
328	42CrMo4	0.38	0.23	0.64	0.99	0.16	0.08	16	1050	10	3.0	0.00	0.00	1.00
329	42CrMo4	0.38	0.23	0.64	0.99	0.16	0.08	16	1050	10	8.8	0.00	0.02	0.98
330	42CrMo4	0.38	0.23	0.64	0.99	0.16	0.08	16	1050	10	51.0	0.00	0.70	0.30
331	42CrMo4	0.38	0.23	0.64	0.99	0.16	0.08	16	1050	10	98.0	0.00	0.80	0.20
332	42CrMo4	0.38	0.23	0.64	0.99	0.16	0.08	16	1050	10	296.4	0.02	0.95	0.03
333	42CrMo4	0.38	0.23	0.64	0.99	0.16	0.08	16	1050	10	611.7	0.04	0.95	0.01
334	42CrMo4	0.38	0.23	0.64	0.99	0.16	0.08	16	1050	10	2010.8	1.00	0.00	0.00
335	42CrMo4	0.38	0.23	0.64	0.99	0.16	0.08	16	1050	10	6961.4	1.00	0.00	0.00
336	50CrMo4	0.50	0.32	0.80	1.04	0.24	0.11	63	1050	10	12.0	0.00	0.00	1.00
337	50CrMo4	0.50	0.32	0.80	1.04	0.24	0.11	63	1050	10	37.4	0.00	0.10	0.90
338	50CrMo4	0.50	0.32	0.80	1.04	0.24	0.11	63	1050	10	110.9	0.00	0.20	0.80
339	50CrMo4	0.50	0.32	0.80	1.04	0.24	0.11	63	1050	10	200.0	0.00	0.60	0.40
340	50CrMo4	0.50	0.32	0.80	1.04	0.24	0.11	63	1050	10	267.3	0.00	0.70	0.30
341	50CrMo4	0.50	0.32	0.80	1.04	0.24	0.11	63	1050	10	1005.2	0.00	0.75	0.25
342	50CrMo4	0.50	0.32	0.80	1.04	0.24	0.11	63	1050	10	2010.8	0.16	0.80	0.04
343	50CrMo4	0.50	0.32	0.80	1.04	0.24	0.11	63	1050	10	6610.4	1.00	0.00	0.00
344	50CrMo4	0.50	0.32	0.80	1.04	0.24	0.11	63	1050	10	61167.0	1.00	0.00	0.00
345	50CrMo4	0.46	0.22	0.50	1.00	0.21	0.22	41	850	10	11.7	0.00	0.01	0.99
346	50CrMo4	0.46	0.22	0.50	1.00	0.21	0.22	41	850	10	35.5	0.00	0.10	0.90
347	50CrMo4	0.46	0.22	0.50	1.00	0.21	0.22	41	850	10	60.9	0.00	0.40	0.60
348	50CrMo4	0.46	0.22	0.50	1.00	0.21	0.22	41	850	10	110.9	0.00	0.70	0.30
349	50CrMo4	0.46	0.22	0.50	1.00	0.21	0.22	41	850	10	281.5	0.00	0.90	0.10
350	50CrMo4	0.46	0.22	0.50	1.00	0.21	0.22	41	850	10	624.5	0.05	0.85	0.10
351	50CrMo4	0.46	0.22	0.50	1.00	0.21	0.22	41	850	10	1080.7	0.25	0.70	0.05
352	50CrMo4	0.46	0.22	0.50	1.00	0.21	0.22	41	850	10	2010.8	0.75	0.24	0.01
353	50CrMo4	0.46	0.22	0.50	1.00	0.21	0.22	41	850	10	7881.9	1.00	0.00	0.00
354	50CrMo4	0.46	0.22	0.50	1.00	0.21	0.22	44	1050	10	4.3	0.00	0.00	1.00
355	50CrMo4	0.46	0.22	0.50	1.00	0.21	0.22	44	1050	10	12.6	0.00	0.01	0.99
356	50CrMo4	0.46	0.22	0.50	1.00	0.21	0.22	44	1050	10	35.5	0.00	0.02	0.98
357	50CrMo4	0.46	0.22	0.50	1.00	0.21	0.22	44	1050	10	57.8	0.00	0.30	0.70
358	50CrMo4	0.46	0.22	0.50	1.00	0.21	0.22	44	1050	10	116.8	0.00	0.55	0.45
359	50CrMo4	0.46	0.22	0.50	1.00	0.21	0.22	44	1050	10	296.4	0.00	0.90	0.10
360	50CrMo4	0.46	0.22	0.50	1.00	0.21	0.22	44	1050	10	1080.7	0.01	0.95	0.04
361	50CrMo4	0.46	0.22	0.50	1.00	0.21	0.22	44	1050	10	2010.8	0.09	0.90	0.01
362	50CrMo4	0.46	0.22	0.50	1.00	0.21	0.22	44	1050	10	7331.1	1.00	0.00	0.00
363	27MnCrV4	0.24	0.21	1.06	0.79	0.02	0.18	14	880	10	2.0	0.00	0.01	0.99
364	27MnCrV4	0.24	0.21	1.06	0.79	0.02	0.18	14	880	10	2.7	0.00	0.07	0.93
365	27MnCrV4	0.24	0.21	1.06	0.79	0.02	0.18	14	880	10	6.7	0.00	0.20	0.80
366	27MnCrV4	0.24	0.21	1.06	0.79	0.02	0.18	14	880	10	11.2	0.01	0.30	0.69
367	27MnCrV4	0.24	0.21	1.06	0.79	0.02	0.18	14	880	10	16.0	0.01	0.70	0.29
368	27MnCrV4	0.24	0.21	1.06	0.79	0.02	0.18	14	880	10	52.3	0.40	0.50	0.10
369	27MnCrV4	0.24	0.21	1.06	0.79	0.02	0.18	14	880	10	83.1	0.60	0.30	0.10
370	27MnCrV4	0.24	0.21	1.06	0.79	0.02	0.18	14	880	10	150.9	0.73	0.17	0.10
371	27MnCrV4	0.24	0.21	1.06	0.79	0.02	0.18	14	880	10	244.6	1.00	0.00	0.00
372	27MnCrV4	0.24	0.21	1.06	0.79	0.02	0.18	14	880	10	719.7	1.00	0.00	0.00
373	27MnCrV4	0.24	0.21	1.06	0.79	0.02	0.18	14	880	10	7271.2	1.00	0.00	0.00
374	27MnCrV4	0.24	0.21	1.06	0.79	0.02	0.18	17	1050	10	2.1	0.00	0.00	1.00

375	27MnCrV4	0.24	0.21	1.06	0.79	0.02	0.18	17	1050	10	4.2	0.00	0.01	0.99
376	27MnCrV4	0.24	0.21	1.06	0.79	0.02	0.18	17	1050	10	9.6	0.00	0.25	0.75
377	27MnCrV4	0.24	0.21	1.06	0.79	0.02	0.18	17	1050	10	14.5	0.00	0.60	0.40
378	27MnCrV4	0.24	0.21	1.06	0.79	0.02	0.18	17	1050	10	55.1	0.01	0.95	0.04
379	27MnCrV4	0.24	0.21	1.06	0.79	0.02	0.18	17	1050	10	102.1	0.05	0.94	0.01
380	27MnCrV4	0.24	0.21	1.06	0.79	0.02	0.18	17	1050	10	154.0	0.12	0.87	0.01
381	27MnCrV4	0.24	0.21	1.06	0.79	0.02	0.18	17	1050	10	332.9	0.30	0.69	0.01
382	27MnCrV4	0.24	0.21	1.06	0.79	0.02	0.18	17	1050	10	757.6	1.00	0.00	0.00
383	27MnCrV4	0.24	0.21	1.06	0.79	0.02	0.18	17	1050	10	7271.2	1.00	0.00	0.00
384	27MnCrV4	0.24	0.21	1.06	0.79	0.02	0.18	17	1050	10	43939.7	1.00	0.00	0.00
385	50CrV4	0.55	0.22	0.98	1.02	0.00	0.01	25	880	5	12.6	0.00	0.00	1.00
386	50CrV4	0.55	0.22	0.98	1.02	0.00	0.01	25	880	5	21.2	0.00	0.01	0.99
387	50CrV4	0.55	0.22	0.98	1.02	0.00	0.01	25	880	5	35.5	0.00	0.07	0.93
388	50CrV4	0.55	0.22	0.98	1.02	0.00	0.01	25	880	5	56.6	0.00	0.45	0.55
389	50CrV4	0.55	0.22	0.98	1.02	0.00	0.01	25	880	5	77.2	0.00	0.80	0.20
390	50CrV4	0.55	0.22	0.98	1.02	0.00	0.01	25	880	5	116.8	0.45	0.35	0.20
391	50CrV4	0.55	0.22	0.98	1.02	0.00	0.01	25	880	5	241.0	1.00	0.00	0.00
392	50CrV4	0.55	0.22	0.98	1.02	0.00	0.01	25	880	5	974.5	1.00	0.00	0.00
393	50CrV4	0.55	0.22	0.98	1.02	0.00	0.01	25	880	5	6961.4	1.00	0.00	0.00
394	50CrV4	0.55	0.22	0.98	1.02	0.00	0.01	25	880	5	67836.1	1.00	0.00	0.00
395	50CrV4	0.55	0.22	0.98	1.02	0.00	0.01	43	1050	5	10.3	0.00	0.00	1.00
396	50CrV4	0.55	0.22	0.98	1.02	0.00	0.01	43	1050	5	19.1	0.00	0.03	0.97
397	50CrV4	0.55	0.22	0.98	1.02	0.00	0.01	43	1050	5	32.0	0.00	0.05	0.95
398	50CrV4	0.55	0.22	0.98	1.02	0.00	0.01	43	1050	5	73.3	0.00	0.25	0.75
399	50CrV4	0.55	0.22	0.98	1.02	0.00	0.01	43	1050	5	143.6	0.10	0.70	0.20
400	50CrV4	0.55	0.22	0.98	1.02	0.00	0.01	43	1050	5	312.2	1.00	0.00	0.00
401	50CrV4	0.55	0.22	0.98	1.02	0.00	0.01	43	1050	5	1080.7	1.00	0.00	0.00
402	50CrV4	0.55	0.22	0.98	1.02	0.00	0.01	43	1050	5	6961.4	1.00	0.00	0.00
403	50CrV4	0.55	0.22	0.98	1.02	0.00	0.01	43	1050	5	67836.1	1.00	0.00	0.00
404	50CrV4	0.47	0.35	0.82	1.20	0.00	0.04	60	880	5	8.0	0.00	0.00	1.00
405	50CrV4	0.47	0.35	0.82	1.20	0.00	0.04	60	880	5	20.5	0.00	0.05	0.95
406	50CrV4	0.47	0.35	0.82	1.20	0.00	0.04	60	880	5	32.0	0.00	0.10	0.90
407	50CrV4	0.47	0.35	0.82	1.20	0.00	0.04	60	880	5	60.2	0.00	0.30	0.70
408	50CrV4	0.47	0.35	0.82	1.20	0.00	0.04	60	880	5	81.3	0.01	0.40	0.59
409	50CrV4	0.47	0.35	0.82	1.20	0.00	0.04	60	880	5	123.0	0.10	0.35	0.55
410	50CrV4	0.47	0.35	0.82	1.20	0.00	0.04	60	880	5	228.8	1.00	0.00	0.00
411	50CrV4	0.47	0.35	0.82	1.20	0.00	0.04	60	880	5	906.4	1.00	0.00	0.00
412	50CrV4	0.47	0.35	0.82	1.20	0.00	0.04	60	880	5	9016.9	1.00	0.00	0.00
413	50CrV4	0.47	0.35	0.82	1.20	0.00	0.04	60	880	5	71438.5	1.00	0.00	0.00
414	50CrV4	0.47	0.35	0.82	1.20	0.00	0.04	26	1050	5	8.6	0.00	0.00	1.00
415	50CrV4	0.47	0.35	0.82	1.20	0.00	0.04	26	1050	5	13.3	0.00	0.01	0.99
416	50CrV4	0.47	0.35	0.82	1.20	0.00	0.04	26	1050	5	37.4	0.00	0.03	0.97
417	50CrV4	0.47	0.35	0.82	1.20	0.00	0.04	26	1050	5	53.7	0.00	0.20	0.80
418	50CrV4	0.47	0.35	0.82	1.20	0.00	0.04	26	1050	5	123.0	0.00	0.40	0.60
419	50CrV4	0.47	0.35	0.82	1.20	0.00	0.04	26	1050	5	312.2	0.06	0.75	0.19
420	50CrV4	0.47	0.35	0.82	1.20	0.00	0.04	26	1050	5	425.8	0.11	0.80	0.09
421	50CrV4	0.47	0.35	0.82	1.20	0.00	0.04	26	1050	5	974.5	1.00	0.00	0.00
422	50CrV4	0.47	0.35	0.82	1.20	0.00	0.04	26	1050	5	7881.9	1.00	0.00	0.00
423	50CrV4	0.47	0.35	0.82	1.20	0.00	0.04	26	1050	5	75232.3	1.00	0.00	0.00

[1] Higher carbon content relative to standard carbon content in steel.
